# Syndromic surveillance and heat wave morbidity: a pilot study based on emergency departments in France

**DOI:** 10.1186/1472-6947-9-14

**Published:** 2009-02-20

**Authors:** Loïc Josseran, Nadège Caillère, Dominique Brun-Ney, Jean Rottner, Laurent Filleul, Gilles Brucker, Pascal Astagneau

**Affiliations:** 1 French Institute for Public Health Surveillance, Saint Maurice, France; 2 Assistance Publique des Hôpitaux de Paris, Paris, France; 3 Emergency Department, Mulhouse general hospital, Mulhouse, France; 4 Bordeaux Regional centre, French Institute for Public Health Surveillance, Bordeaux, France; 5 Department of public health, Pierre et Marie Curie University School of Medicine, Paris, France

## Abstract

**Background:**

The health impacts of heat waves are serious and have prompted the development of heat wave response plans. Even when they are efficient, these plans are developed to limit the health effects of heat waves. This study was designed to determine relevant indicators related to health effects of heat waves and to evaluate the ability of a syndromic surveillance system to monitor variations in the activity of emergency departments over time. The study uses data collected during the summer 2006 when a new heat wave occurred in France.

**Methods:**

Data recorded from 49 emergency departments since July 2004, were transmitted daily via the Internet to the French Institute for Public Health Surveillance. Items collected on patients included diagnosis (ICD10 codes), outcome, and age. Statistical t-tests were used to compare, for several health conditions, the daily averages of patients within different age groups and periods (whether 'on alert' or 'off alert').

**Results:**

A limited number of adverse health conditions occurred more frequently during hot period: dehydration, hyperthermia, malaise, hyponatremia, renal colic, and renal failure. Over all health conditions, the total number of patients per day remained equal between the 'on alert' and 'off alert' periods (4,557.7/day vs. 4,511.2/day), but the number of elderly patients increased significantly during the 'on alert' period relative to the 'off alert' period (476.7/day vs. 446.2/day p < 0.05).

**Conclusion:**

Our results show the interest to monitor specific indicators during hot periods and to focus surveillance efforts on the elderly. Syndromic surveillance allowed the collection of data in real time and the subsequent optimization of the response by public health agencies. This method of surveillance should therefore be considered as an essential part of efforts to prevent the health effects of heat waves.

## Background

Over the last 30 years, the impact of major heat wave shocks on population morbidity and mortality has become an urgent public health concern [1-3]. In different countries, stakeholders and health authorities are often overwhelmed by the magnitude of both the threat to public health and the social impact of heat waves. This reflects a lack of preparation and failed organization within the healthcare system. Public awareness campaigns can be oriented to promotion of the adoption of various preventive measures, such as the creation of air-conditioned areas in nursing homes and active hydration programs for high-risk groups such as the elderly or young children. Nevertheless, the first step in preventing the drastic consequences of such climatic events as heat waves is to implement systems to provide early warning based on prompt detection of excess morbidity and mortality [4]. Such an early-warning surveillance system should examine data in real time and it should be highly sensitive and cost-effective.

In August 2003, France suffered an extreme heat wave that had major public health consequences. Overall 15,000 extra deaths during a 16-day period were recorded, corresponding to a 60% increase in expected mortality [5,6]. Since summer 2004, the French Institute for Public Health Surveillance, in close cooperation with Météo France^® ^defined and implemented a heat health watch warning system on the basis of biometerological indicators. The warning system operates from 1^st ^June to 31^st ^August (level 1, seasonal surveillance period). When the alert criteria are fulfilled, an awareness and action level is declared by the *Préfet *(level 2) who manages the *département *(French administrative unit). A third level (maximum mobilization) is implemented if the impacts of heat wave overwhelm the health field: power cuts, drought, management problems in the funeral centres and heavy air pollution.

The alert system aims to give the public authorities 3 days' prior warning that a heat wave may occur, in order for the National Heat Wave Plan (NHWP) measures to be put into operation. The preventive measures are aimed at modifying the behaviour of people, health institution and health authorities with regard to high summer temperatures [7,8].

The health impact of hot weather is measured in routine during the seasonal surveillance period (from 1^st ^June to 31^st ^August) on the basis of the rough daily activities of emergency departments in hospitals (ED) and mortality data [7].

In the summer of 2006, a new heat wave occurred that lasted approximately three weeks, and generated an excess mortality of 2,100 deaths [8,9]. This extreme heat event has been detected by the national heat/health watch warning system which has been able to identify properly hot days on the basis of 3 day prediction temperatures [9].

Using data collected during the 2006 heat wave in France, we undertook this study to evaluate the ability of a syndromic surveillance system to detect health impacts of heat wave through variations in ED activity over time and to determine relevant indicators for this kind of surveillance in this particular weather situation.

## Methods

### Data collection

Following the 2003 heat wave, a volunteer surveillance network of 49 hospital emergency departments (ED) was set up to collect individual patient data on a daily basis. This system is called Oscour^® ^"Organisation de la Surveillance Coordonnée des Urgences" (Coordinated Health Surveillance of Emergency Departments). Details of the network have already been published [10]. The 49 hospitals involved in the network correspond to 9.8% of the daily ED activity in France. The present study analyzed the following data for individual patients collected through this network: age, reason for emergency admission, primary medical diagnosis according to the physician at ED discharge (based on the tenth edition of the International Classification of Diseases (ICD-10)), and whether the patient was admitted for hospitalisation after ED discharge. These data had been transmitted in encrypted form to the French Institute for Public Health Surveillance; every day, the data from the preceding 24 hours (midnight to midnight) were sent over the Internet using FTP (File Transfer Protocol). All hospital discharge records were anonymized and handled in accordance with national rules of confidentiality.

Temperature data were obtained from the French Weather Bureau (Météo France^®^). The data contained the daily values of maximum and minimum temperatures (°C) measured by a network of 22 regional weather stations. The daily national mean temperature was calculated on this basis following the methods of Météo France^® ^[9].

### Definitions of heat wave and alert period

The current study was conducted between June 1^st ^and August 31^st^, 2006, and it used criteria defined in the NHWP [11]. Alert thresholds were defined based on the relationship between mortality and maximum and minimum temperatures over three consecutive days during a 30-year period. The thresholds were 50% excess mortality in large metropolitan areas and 100% in other cities. The threshold was calculated for 100 cities in France corresponding to one city for each French administrative *département *and stand for the *département *[11]. The heat wave period was defined to include days when the *département *alert threshold was exceeded in at least one *département *in France. The period included 18 days from July 11 to July 28, 2006; this constituted the 'on alert' period (ONAP) and it involved 60 *départements *of 96 in France [12]. The 'off alert' periods (OFAP) were defined as June 1 to July 10 and July 29 to August 31 corresponding to 74 days.

### Definition of syndromes and age groups and statistical analysis

The age groups were defined as younger adults (< 75 years old) and elderly adults (≥ 75 years old) and based on a literature review, syndromes were defined according to the corresponding ICD10 codes (Table 1) [13-18]. A comparison of the daily percentage of diagnosis miscoded or uncoded during ONAP and OFAP has been done.

**Table 1:** ICD 10 codes used for syndrome selection

**Syndromes**:	ICD 10 Codes
**Hypoglycemia**:	E162

**Dehydration**:	E86

**Hyponatremia/Hypo-Osmolality**:	E871

**Diseases of the circulatory system**:	I00 → I99

**Cerebrovascular diseases**:	I60 → I69

**Diseases of the respiratory system**: (Exempt J45–J46)	J00 → J99

**Asthma**:	J45: Asthma
	J46: Status asthmaticus

**Urinary Infections**:	N10: Acute tubulo-interstitial nephritis
	N30: Cystitis
	N34: Urethritis and urethral syndrome
	N151: Renal and perinephric abscess
	N390: Urinary tract infection, site not specified
	N410: Acute prostatitis

**Renal failure**:	N17: Acute renal failure
	N18: Chronic renal failure
	N19: Unspecified renal failure

**Renal Colic**:	N20: Calculus of kidney and ureter
	N21: Calculus of lower urinary tract
	N22: Calculus of urinary tract in diseases classified elsewhere
	N23: Unspecified renal colic

**Malaise**:	R42: Dizziness and giddiness
	R53: Malaise and fatigue
	R55: Syncope and collapse

**Hyperthermia**:	T67: Effects of heat and light
	X30: Exposure to excessive natural heat

**Figure 1:**
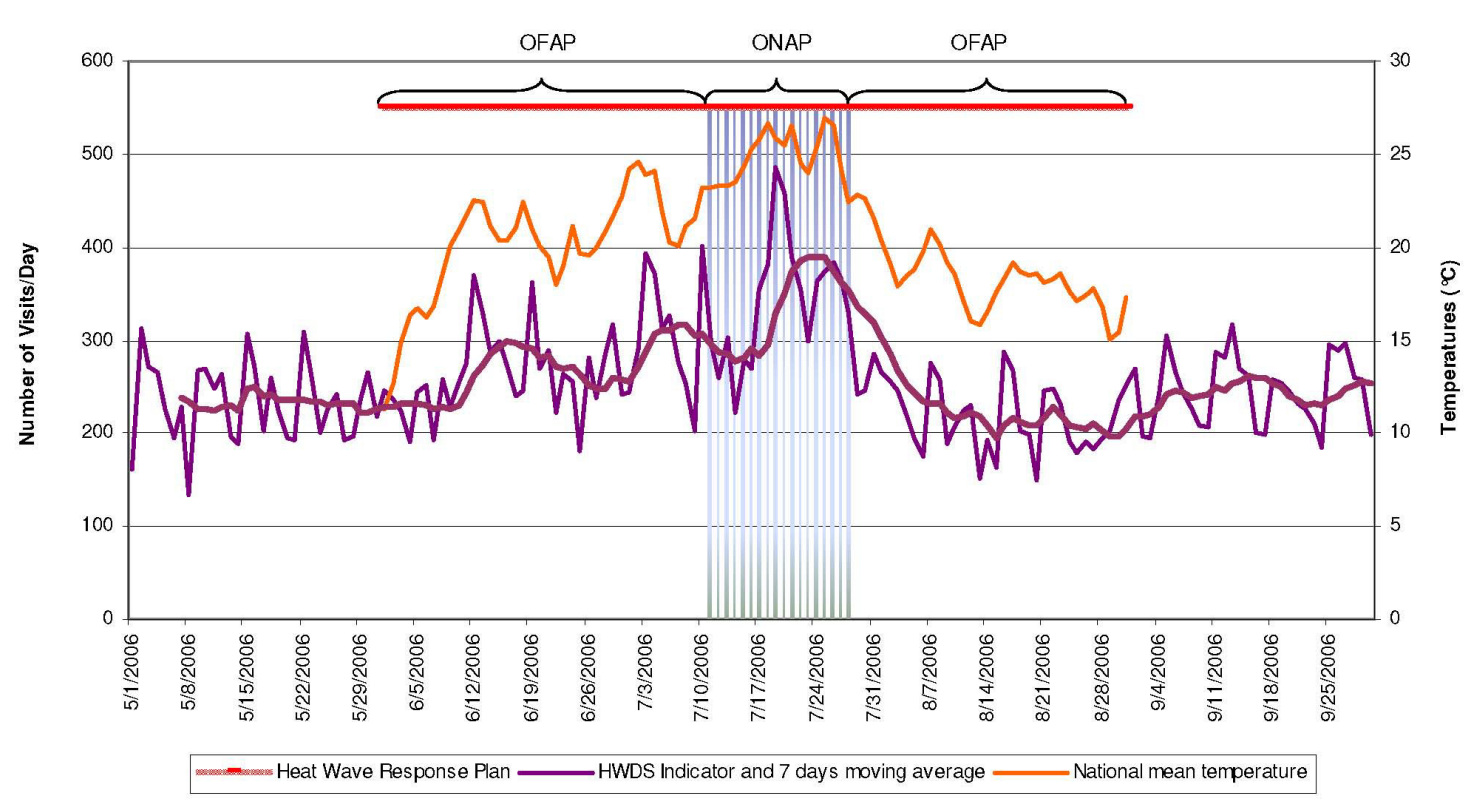
**Evolution of the heat wave disease syndrome indicator and the national mean temperature 49 ED, France, 2006**.

The daily average numbers of ED visits and hospitalisation outcomes for each syndrome and for each age group were compared between ONAP and OFAP. A group of heat wave disease syndromes (HWDS) was defined to include those diagnoses that were significantly more frequent during ONAP than during OFAP. The HWDS indicator based on this group is presented in Figure 1 and is compared with the national temperature recorded by Météo France^®^. ED visits for other diagnoses were also included in the analysis. Comparisons of proportions and means were done using the Pearson chi square test and Student's t-test. Differences were considered significant when p < 0.05. Statistical analyses were carried out using the Statistical Package for the Social Sciences version 13 program (SPSS^® ^v13).

### Ethical approval

The use of this database in the frame of epidemiological studies has been authorized by the French National Commission for Data protection and the Liberties (CNIL) and has received an agreement number: 1015929 in accordance with the Act n°78-17 of 6 January 1978 on Data Processing, Data Files and Individual Liberties.

## Results

From the 1^st ^of June to the 31^st ^of August 2006, 415,862 visits were logged in the system. Among them, 82,040 (19.7%) were recorded during ONAP, including 8,580 (10.5%) patients aged 75 years and older, and 15,996 (19.5%) children aged 15 or younger. A total of 91,802 hospitalisations were recorded, 17,384 (18.9%) of them during the ONAP including 4,991 (28.7%) involving people aged 75 or older. The daily mean number of patients per day was not significantly different between the ONAP and OFAP across both age groups (4,557.7/day vs. 4,511.2/day, respectively (Table 2)). The number of venues per day was, however, significantly different for elderly people (476.7/day vs. 446.2/day, p < 0.05). Similar results were observed for the daily mean number of hospitalisations: hospitalisation outcomes including both age groups were 1,011.6/day during ONAP vs. 994.6/day during OFAP (NS); for elderly people: 257.6/day vs. 227.7/day, p < 0.05). The ratio of males to females during ONAP across both age groups was 1.2, and this number did not change significantly between ONAP and OFAP. The daily percentage of diagnosis miscoded or uncoded was not significantly different between ONAP and OFAP (23.7% vs. 24.2% respectively)

**Table 2:** Comparison of daily mean number of visits/hospitalisations ('on' and 'off alert') 49 ED, France, 2006

Number of/Period	ONAP^1^	OFAP^2^	p
**Patient Visit**	4557.7	4511.2	NS

**Hospitalized Patient**	1011.6	994.6	NS

**Malaise**	137.2	102.9	< 0.001

**Hyperthermia**	7.1	1.7	< 0.001

**Hyponatremia**	11	3.9	< 0.001

**Dehydration**	12.6	4.3	< 0.001

**Hypoglycemia**	7.3	7.7	NS

**Urinary Infection**	34.6	35.2	NS

**Renal Colic**	39.1	34.9	< 0.05

**Diseases of the respiratory system (except asthma)**	162.4	165.6	NS

**Asthma**	31.9	31.7	NS

**Diseases of the circulatory system**	104.9	109.5	NS

**Cerebrovascular disease**	21.0	21.6	NS

**Renal failure**	6.9	5.1	0.05

**Deaths in ED**	4	3.5	NS

1: ONAP = 'On Alert' Period; 2: OFAP = 'Off Alert' Period.

Patients presented with malaises, dehydration, hyperthermia, hyponatremia, renal colic, or renal failure, were observed at significantly higher frequency during ONAP than OFAP (Table 2). In contrast, neither the number of deaths recorded in ED nor the number of patients diagnosed with respiratory nor cerebrovascular diseases differed significantly between the two time periods (Table 2). Therefore, the group of HWDS was composed of the following diseases: malaises, dehydration, hyperthermia, hyponatremia, renal colic and renal failure (Table 2 and Table 1 for ICD-10 codes). Figure 1 displays the indicator derived from the HWDS, the national average temperature observed in France during ONAP and OFAP, and the NHWP. The figure shows that the indicator increases one day after every increase in temperature.

ED visits and hospitalisations increased significantly for the elderly during ONAP, relative to OFAP (10.5% vs. 9.9% and 28.7% vs. 25.5%, respectively, p-value for both differences < 0.001; Table 3). Higher proportions of the elderly were admitted to the ED or were hospitalized during ONAP compared to OFAP in relation to a HWDS (27.7% vs. 23.8% and 58.0% vs. 50.1%, respectively, p-value for both differences p < 0.001; Table 3).

**Table 3:** Total ED visits, hospitalisations, and heat wave diseases syndromes, 49 ED – France, summer 2006

	ED Visits		Hospitalisations		HWDS^1 ^ED Visits		HWDS^1 ^Hospitalisations	
	
	< 75 years old	≥ 75 years old	< 75 years old	≥ 75 years old	< 75 years old	≥ 75 years old	< 75 years old	≥ 75 years old
**ONAP^2^**	73,460 (89.5%)	8,580*** (10.5%)	12,393 (71.3%)	4,991*** (28.7%)	2,775 (72.3%)	1,068** (27.7%)	453 (42.0%)	626*** (58.0%)

**OFAP^3^**	300,804 (90.1%)	33,018 (9.9%)	55,420 (74.5%)	18,998 (25.5%)	8,612 (76.2%)	2,694 (23.8%)	1,428 (49.1%)	1,436 (50.1%)

**Total**	374,264 (90.0%)	41,598 (10.0%)	67,813 (73.9%)	23,989 (26.1%)	11,387 (75.2%)	3,762 (24.8%)	1,881 (47.7%)	2,062 (52.3%)

* p < 0.05 ** p < 0.01 *** p < 0.001.

1: HWDS, Heat Wave Disease Syndromes; 2: ONAP = 'On Alert' Period; 3: OFAP, 'Off Alert' Period.

As shown in Table 4, the proportion of visits linked to HWDS during the heat wave increased significantly for elderly people: the proportion of total visits linked to HWDS was 12.43% during ONAP compared to 8.15% during OFAP (p < 0.001).

**Table 4:** Heat wave disease syndromes (visits/proportion) by age group and period, 49 ED, France summer 2006

	Less than 75 years old				At least 75 years old			
	**ONAP^1^**		**OFAP^2^**		**ONAP^1^**		**OFAP^2^**	

	HWDS^3 ^ED Visits	HWDS^3 ^ED Visits as a proportion of total ED Visits	HWDS^3 ^ED Visits	HWDS^3 ^ED Visits as a proportion of total ED Visits	HWDS^3 ^ED Visits	HWDS^3 ^ED Visits as a proportion of total ED Visits	HWDS^3 ^ED Visits	HWDS^3 ^ED Visits as a proportion of total ED Visits

**Malaise**	1,808	2.46%***	5,554	1.85%	661	7.70%***	2,062	6.24%

**Hyperthermia**	98	0.13%*	115	0.04%	31	0.35%***	10	0.03%

**Hyponatremia**	59	0.08% (NS)	99	0.03%	139	1.62%***	187	0.57%

**Dehydration**	67	0.09% (NS)	112	0.04%	161	1.88%***	207	0.63%

**Renal Colic**	684	0.94%*	2,527	0.84%	19	0.22% (NS)	58	0.17%

**Renal failure**	59	0.08% (NS)	205	0.07%	57	0.66%*	170	0.51%

**Total HWDS^3^**	2,775	3.78%***	8,612	2.87%	1,068	12.43%***	2,694	8.15%

* p < 0.05 ** p < 0.01 *** p < 0.001.

1: ONAP, 'On Alert' Period; 2: OFAP, 'Off Alert' Period; 3: HWDS, Heat Wave Disease Syndromes

Three diseases increased dramatically during ONAP for this specific age group: hyperthermia (11.5-fold increase), hyponatremia (2.8-fold), and dehydration (3.0-fold). For younger adults, the proportion of total visits related to HWDS was 3.78% during ONAP compared to 2.87% during OFAP (p < 0.001). Only diagnoses presented in Table 4 increased significantly between the two time periods.

## Discussion and conclusion

This study documents the first use of syndromic surveillance based on hospital emergency department data to observe and analyze in detail the public health burden of a major heat wave. The results show that a limited number of indicators are possibly useful for daily monitoring of the health impact of a heat wave on a population using syndromic surveillance. The results also show that the elderly are more severely affected by hot weather than young people. This system provides qualitative and quantitative elements for understanding the immediate public health danger and to evaluate it in real time. The parameters monitored by this system of environmental health surveillance are crucial components because weather modifications require permanent state of adaptability of public health responses, as shown in Figure 1[19,20].

During the OFAP we observed a correlation between the HWDS indicator and temperature peaks in mid June and the beginning of July. But during the ONAP the health effects of the heat wave only appear 3–4 days after the heat wave has begun. This lag may be due to physiological adaptation and/or preventive measures stipulated by the national heat wave plan and activated during the heat wave [7]. The non increase in our indicator during the first days of the "official" beginning of the heat wave made possible such interpretation. This hypothesis should be confirmed by new studies conducted in similar context: a heat wave occurring while a heat wave plan is activated.

During the 'on alert' period, the percentage of patients admitted to hospital emergency departments who were elderly was three times greater than the percentage who were younger adults. Nevertheless, the number of ED admissions across the nation during the heat wave did not change significantly from normal levels. This finding is consistent with other studies, which suggests the necessity of monitoring ED activities for different age groups and of collecting medical diagnosis in real time [21]. It is known that cardiovascular mortality increased during a heat wave [2,22]. Also in agreement with other findings, our results show that hot weather does not affect cardiovascular morbidity [14,23]. This point is discussed in the literature and an impact on cardiovascular morbidity has been shown by others in case of extreme temperatures [24]. In our study, we did not find any significant increase in several other diseases frequently associated with hot weather, such as respiratory diseases, asthma (despite that several cities announced air pollution alerts during the heat wave), urinary infections, hypoglycaemia, or cerebrovascular diseases [1,14].

In terms of number of visits and hospitalisations, elderly patients (≥ 75 years) were the only group to show a significant increase during the 'on alert' period compared to the 'off alert' period. As a result, we think that effort to monitor the health burden of a heat wave should focus on elderly people and on the following diseases in that age group: malaise, hyperthermia, hyponatremia, and dehydration. An increase in one of these parameters, in case of hot weather, is likely to indicate the onset of health effects of a high temperature and it therefore could constitute a signal to initiate a rapid public health response.

This study used several ICD-10 codes to define syndromes relevant to detecting the health impacts of heat waves. This approach was motivated by the concern that the codes are too fine-grained for detection of syndromes related to heat waves. Development of the nationwide surveillance network required accurate coding, inter-coder reliability, and minimal variation in coding over time. By clustering codes, we hope to include all codings that may conceivably be applied to a patient exhibiting some relatively common syndromes related to high temperature [25].

Our study is limited in several ways. The geographical coverage of the network during the study was limited to large cities and so is not entirely representative of the French population. Because most heat effects are more significant in cities with their own "island heat effect areas", similar effect might be expected in other cities. For other areas (i.e., rural area), the impact of heat wave on population could be different and complementary analysis should be necessary. Since the complete automation of data transmission is crucial, not all emergency departments record all of the information per visit which creates an information deficit in the health surveillance and alert system. Given their pivotal role in public health surveillance, ED health care professionals should be trained in collecting and validating data in ways that maximize the quality of surveillance. Our results are supported by the analysis of a single heat wave and should be confirmed by other studies in the same weather conditions. An over estimation of our findings is possible due to over-diagnosis by doctors aware that a heat wave has been declared. In such situation ED physicians are more attentive to heat effects and may code first heat related syndromes. This effect should be taken in account and might explain a part of the increase in some diagnoses.

It is now well accepted that ED activity reflects the overall state of health of a population [26]. ED activity should therefore be considered as a public health 'sentinel' indicator, and health surveillance of EDs should be used to design appropriate public health interventions [27]. Because this method of surveillance uses routinely generated data and does not require the health care professionals to enter additional information, there would be minimal disruption to the work patterns of the data providers [28].

Until now in case of heat wave, weather forecast allowed understanding what would happen in the following days, the health impact of a heat was estimated on the basis of the number of deaths observed and the rough number of daily ED visits for the main city of each French *département*. This system, made possible the follow up by age groups targeted by preventive measures (elderly) or not (younger) and their adaptation in real time if necessary. It gives to public health authorities and stakeholders information near real time about the possible impact of temperature on population. In this circumstance, syndromic surveillance based on EDs, which is already accepted as a validated tool for public health surveillance in the field of infectious diseases [29], might occupy a key position to propose extending syndromic surveillance to managing environmental health concerns [27]. If our results are validated by new studies, applying syndromic surveillance to environmental health concerns may be considered by the public health community. Further studies are now necessary to evaluate the efficacy of this type of surveillance in preventing the public health effects of heat waves.

## Abbreviations

ONAP: On Alert Period; OFAP: Off Alert Period; ED: Emergency Department; ICD-10: International Classification of Diseases 10^th ^revision; HWDS: Heat Wave Disease Syndromes; NHWP: National Heat Wave Plan.

## Competing interests

The authors declare that they have no competing interests.

## Authors' contributions

All authors contributed to project conception. LJ was in charge of the development of the network and epidemiological data analysis, improvement of the ED – Syndromic surveillance system. NC worked on computer programming for all data analysis. DBN was in charge of the regional data collection in Paris area, defined the syndromes groupings and helped to draft the manuscript. JR, head of the Emergency Department of Mulhouse General Hospital, helped to draft manuscript from the point of view of emergency medicine. LF, head of the InVS regional office of Bordeaux, participated in the design of the study and the redaction of the article. GB, head of the French Institute for Public Health Surveillance, initiated this syndromic surveillance system in France. He's participated to the methods definition and helped to draft manuscript. PA, head of the Department of public health, Pierre et Marie Curie University School of Medicine, Paris, France, participated to the definition of methods and article redaction. All authors read and approved the final manuscript.

## Pre-publication history

The pre-publication history for this paper can be accessed here:

http://www.biomedcentral.com/1472-6947/9/14/prepub
